# Blood Pressure and Self-management in Black Women With Hypertension: Protocol Revisions to the Brain Relationships Among Information, Neuroprocessing, and Self-Management Study Due to the COVID-19 Pandemic

**DOI:** 10.2196/43849

**Published:** 2023-04-27

**Authors:** Lenette M Jones, Kayla de Marco, Katharine Keener, Korrey E Monroe

**Affiliations:** 1 Health Behavior and Biological Sciences School of Nursing University of Michigan Ann Arbor, MI United States; 2 School of Nursing University of Michigan Ann Arbor, MI United States

**Keywords:** Black, BRAINS, COVID-19 pandemic, eHealth, Facebook, hypertension, protocol, videoconferencing, web-based, women

## Abstract

**Background:**

The COVID-19 pandemic and the halt to in-person research activities beginning in March 2020 brought new challenges to protocol development and implementation. Due to the pandemic, we had to revise our protocol for the Brain Relationships Among Information, Neuroprocessing, and Self-Management (BRAINS) study, which was designed to examine health information behavior, brain activity, diabetes status, and self-management behavior among Black women with hypertension.

**Objective:**

This report outlines 7 steps describing how our research team (1) revised the BRAINS study protocol, (2) implemented a remote method of data collection, and (3) mitigated the challenges we faced.

**Methods:**

Prior to March 2020, Black women with hypertension were invited to participate in the BRAINS study to undergo a functional magnetic resonance imaging scan, complete surveys, have their blood pressure measured, and have their blood drawn. After these measures were collected, participants would receive phone calls from a dietician to complete two 24-hour dietary recalls using the Nutrition Data System for Research. Our revised protocol relied on a web-based, interactive approach. Participants received a study kit that included an Omron automatic home blood pressure monitor and a hemoglobin A_1c_ kit from the DTIL laboratory. In a Zoom meeting with each participant, our team played an introductory video, administered surveys (via Qualtrics), and guided participants through measuring their blood pressure and performing a finger stick to collect a blood sample for hemoglobin A_1c_ testing. We examined cognitive function using the TestMyBrain Digital Neuropsychology Toolkit, as we were unable to access the functional magnetic resonance imaging laboratory to assess brain activity. The 7 steps in revising our protocol were as follows: conceptualizing the move from in-person to remote study activities (step 1); contacting the funders (step 2); submitting changes for Institutional Review Board approval (step 3); preparing to implement the revised protocol (step 4); implementing the study changes (step 5); mitigating challenges (step 6); and evaluating protocol implementation (step 7).

**Results:**

Approximately 1700 individuals responded to web-based advertisements about the BRAINS study. A total of 131 individuals completed our eligibility screener. We conducted our first Zoom appointment in July 2020 and our last Zoom appointment in September 2020. Using our revised strategies, a total of 99 participants completed all study measures within a 3-month period.

**Conclusions:**

In this report, we discuss successes and challenges in revising our protocol and reaching our population of interest remotely, safely, and effectively. The information we have outlined can help researchers create similar protocols to reach and conduct research remotely with diverse populations, such as individuals who are unable to participate in studies in person.

**International Registered Report Identifier (IRRID):**

DERR1-10.2196/43849

## Introduction

The COVID-19 pandemic complicated the delivery of medical care and the implementation of research studies [1]. While health assessments typically occur in person and include blood pressure readings and other tests conducted by a trained professional, these standards of care were not feasible given the “Stay at Home” orders and imminent dangers of being in-person for routine health care appointments. During the beginning of the pandemic, there was a significant decrease in the “usual care” of preventative screening and monitoring of chronic illnesses [2,3].

Consequently, a shift to self-reported data emerged after March 2020. Although self-reported data has the benefit of being easier to obtain at a smaller expense, there are some considerable drawbacks. These include some participants’ inability and willingness to recall and report their measurements [4]. Factors such as age, formal education level, and regular access to health care professionals impact the accuracy of self-reporting, especially self-reporting of hypertension status [5]. Given barriers to accessing health care services at the beginning of the COVID-19 pandemic, the discrepancies related to self-reported data were even more concerning, particularly among vulnerable groups like Black women with hypertension.

Hypertension affects approximately 57.6% of Black women in the United States [6]. Black women who have hypertension are also more likely to have higher blood pressure levels, which increases the risk for other chronic illnesses, such as end-stage renal disease and type 2 diabetes [7]. While hypertension and type 2 diabetes frequently co-occur among all racial and ethnic groups, Black women’s odds of this comorbidity are 6 times higher than the odds for White women [8]. Additionally, Black women are the most likely of any racial group to have undiagnosed diabetes [9].

The decrease in usual care at the beginning of the pandemic was even more concerning for individuals who were already part of vulnerable groups, such as Black women with hypertension. Previously, some health care professionals reported difficulty in reaching diverse populations, and this was magnified during the pandemic [10-12]. Prior to the pandemic, previous studies highlighted the barriers to reaching Black individuals with chronic illnesses [10,11]. Not only were Black individuals more severely affected by COVID-19 [12-14], those with chronic illnesses were more likely to have increased difficulty in managing them [15,16]. Previous studies highlight the viability of videoconferencing platforms as tools to meet with patients, but state that additional studies are needed to further assess their utility [17,18].

Prior to the COVID-19 pandemic, we developed an in-person protocol (Brain Relationships Among Information, Neuroprocessing, and Self-Management [BRAINS]) to assess the relationships among health information behavior, brain activity and executive function, diabetes status, and health outcomes among Black women with hypertension. However, to ensure the safety of our participants and our research team, we had to move to remote data collection during the COVID-19 pandemic. In this report, we outline the following seven steps we completed in modifying the BRAINS study: (1) conceptualizing how to move from in-person to remote, (2) contacting funders, (3) submitting Institutional Review Board (IRB) changes, (4) preparing to implement our revised protocol, (5) implementing our protocol, (6) mitigating the challenges we faced, and (7) evaluating our implementation. This study was novel not only because we were able to quickly pivot to remote data collection, but we also were able to reach our diverse population of interest (Black women with hypertension), which is traditionally regarded as a “difficult to reach” group [19]. Future studies can use these guidelines to develop thoughtful, informed, evidence-based, and carefully crafted protocols to reach other diverse or underrepresented populations.

## Methods

### Study Overview

The BRAINS study aimed to examine relationships among health information behavior, brain activity, diabetes status, and self-management behavior among Black women with hypertension. The study design was descriptive, cross-sectional, and measured concepts using surveys, blood pressure readings, serum testing, and functional magnetic resonance imaging (fMRI) scans (see Textbox 1 for a list of measures). After receiving IRB approval, study recruitment began in June 2020. We used a Facebook campaign to seek individuals who self-identified as Black women. Additional inclusion criteria were being 21-64 years of age, being diagnosed with hypertension by a health care professional, having access to a computer or tablet, and living in the metro Detroit area (determined by zip code).

**Brain Relationships Among Information, Neuroprocessing, and Self-Management study measures.**

Measures:

- Health information behavior [20]
- Neuroprocessing using functional magnetic resonance imaging [21]
- Self-efficacy [22]
- Self-regulation [23]
- Patient activation [24]
- Self-management diet behaviors (Nutrition Data System for Research) [25-27]
- Blood pressure [28,29]
- hemoglobin A_1c_(HbA_1c_) [30]
- Quality of life (Patient-Reported Outcomes Measurement Information System) [31]
- Basic cognitive assessment—Montreal Cognitive Assessment [32]

### Ethical Considerations

This study was reviewed and approved by the University of Michigan IRB (HUM00138174). Women who were interested in participating in the study reviewed the BRAINS study consent form, then provided informed consent by clicking “Yes, I consent,” prior to completing study measures in Qualtrics [33]. Each participant was assigned a study ID number. All study data were deidentified and stored according to study ID. After participants completed all study measures, they received US $75 gift card to compensate them for their time.

In this report, we outline the 7 steps that we completed to revise our BRAINS study from an in-person protocol to a fully remote study.

### Steps in Revising the Protocol

#### Step 1: Conceptualizing the Move From In-Person to Remote Study Activities

The COVID-19 pandemic brought all in-person study activities to a complete halt. We had to decide if we were going to suspend our study activities or pivot to a fully web-based format. We asked the following questions:

(1) *How can we meet safely with our participants?* The first decision we made was to use Zoom, a videoconferencing platform, to meet with our participants [17]. We felt that Zoom would be the best web-based platform where we could send links to surveys and assist participants as needed. We were already using Qualtrics as a platform for our surveys, so we were sure that we could continue to use it remotely by sending participants a link.

(2) *Of the remaining measures, which can be collected remotely and which cannot?* In addition to the surveys we planned to deliver via Qualtrics, we still needed to determine how to collect blood pressure readings, serum testing, and fMRI scans, or find alternative measures. See Table 1 for an overview of the original protocol compared to the protocol changes and revisions.

(i) *Blood pressure measurement*. To obtain participants’ blood pressure readings, we selected the Omron 10 series automatic home blood pressure monitor (HBPM). We selected this HBPM for 2 reasons. First, the Omron 10 Series HBPM received satisfactory results during validation testing [34,35]. Second, this HBPM contains a cuff that can accurately obtain blood pressure readings on an arm with a circumference of 9 inches to 17 inches, which is a larger cuff than others included with most HBPM [36]. This allowed for accurate blood pressure readings from a wide variety of participants, and less concern about the diameter of the participant’s arm.

(ii) *Blood glucose measurement*. Our team had a previously established relationship with DTIL, a company that packaged and processed at-home hemoglobin HbA_1c_ tests. Therefore, we send their kits to participants to assess their blood glucose levels [30].

(iii) *Measures we were unable to capture*. We did not find at-home alternative measures for high-sensitivity C-reactive protein collection or the fMRI scans. The cost of doing an at-home high-sensitivity C-reactive protein test was not feasible for this study, and there is no replacement for the brain activity data that can be gathered from an fMRI scan. However, we were able to gather data on cognitive function pathways using Harvard University’s TestMyBrain, which is a web-based battery of neurocognitive tests [37].

(3) *How will we get the new required supplies to our participants?* We decided to mail participants a study welcome kit that included a welcome letter, a HBPM, and a HbA_1c_ test. We chose to use United Parcel Service to mail the kits because their shipping time estimates were the fastest, and we did not want any delays in getting our supplies to our participants. Our university also had an account preregistered with United Parcel Service that would bill directly to our study’s shortcode. All supplies were inventoried before being sent out, and the HbA_1c_ kits were labeled with our study participant ID numbers to make sure that each sample and result would be matched to their other study results.

(4) *How will we obtain informed consent?* We met with our institution’s IRB staff to understand how to prepare for and obtain consent remotely. We were advised that we needed to present the consent form to participants in the same format as we usually would, and they would need to read the consent themselves. Contact information for the study was given in multiple places, in case potential participants had questions about their participation. At the bottom of the consent page, we asked if participants had thoroughly read and agreed to participate in all study activities. They could either check yes or no. If they checked yes, they were able to complete screening questions to confirm their eligibility for the study. If they checked no, they were routed to a screen that thanked them for their time.

**Table 1:** The Brain Relationships Among Information, Neuroprocessing, and Self-management (BRAINS) study protocol—original, revised, and additions.

Measure	BRAINS in-person (original protocol)	BRAINS remote (revised protocol)	Additions to revised protocol
Qualtrics surveys	Completed on an iPad, sitting next to a study team member	Completed during a Zoom appointment, with a study team member	Added 7 questions about changes required to blood pressure self-management routine
fMRI^a^ scan	Completed in the fMRI laboratory	Unable to conduct	None
Cognitive assessment	MoCA^b^	MoCA	TestMyBrain
Blood pressure	Measured by a study team member	Measured by the participant, with guidance from a study team member	None
HbA_1c_^c^	Collected by a research nurse	Collected by the participant, with guidance from a study team member	None
High-sensitivity C-reactive protein	Collected by a research nurse	Unable to collect	None
24-hour dietary recall	Collected by a dietician via telephone	Collected by a dietician via telephone	None

^a^fMRI: functional magnetic resonance imaging.

^b^MoCA: Montreal Cognitive Assessment.

^c^HbA_1c_: hemoglobin A_1c_.

#### Step 2: Contacting the Funders

After we conceptualized the changes required and had evidence to support each change, we were able to reach out to the lead investigators who obtained the funds for this study. There were 2 funding sources to support this study; both sources of funding were National Institute of Health center pilot grants (P20 and P30). We wrote a letter to both center directors, briefly describing the challenges we were facing and requesting a meeting to discuss the changes we wanted to implement. During both meetings (Zoom), we displayed PowerPoint slides with our revised study aims and goals for the safety of both participants and members of our team. After meeting with both center directors, we received feedback that our changes were thoughtful and reasonable; therefore, approval to revise the protocol was granted.

Given that we could not access the fMRI lab and complete the fMRI scans, we had an excess of funds to spend. Included in our letter to the center directors was a request to increase the sample size from 50 to 100 participants. We were also able to increase our study incentive to further encourage participation. Both directors agreed that this request made sense and approved it, so we created an IRB amendment to receive their approval as well.

#### Step 3: Submitting Changes for IRB Approval

We met with a representative from the University’s IRB team to receive guidance on revising our protocol. We wanted to make sure that we were aware of all the new requirements related to the COVID-19 pandemic. We asked questions about remote consent, how other studies implemented revisions to their protocols, and how long we could expect before being notified that our revised amendment was approved. This meeting was critical to our success, as we relied on their expertise to complete the revisions in a timely fashion, and we required IRB approval to move forward with the study. We prepared and submitted our IRB amendment, then waited for approval.

#### Step 4: Preparing to Implement the Revised Protocol

#### Overview

After receiving IRB approval for our revised protocol, there were several tasks to complete to prepare for the implementation of the study. We had to buy supplies, prepare materials to be mailed, establish a contract with the shipping company, and develop a tracking system to make sure that the correct box got to its intended participant. We purchased boxes and sealable plastic shipping bags. This would keep the study materials protected from physical and water damage during the shipping process. To prepare the materials to be mailed to the participants, all study supplies were inventoried, assigned, and labeled with participant study ID numbers to ensure that the correct supplies went to the correct participant. This was particularly important for the HbA_1c_ kits, as the participant would need to send their prelabeled sample back to the DTIL laboratory.

Each participant’s HBPM and HbA_1c_ kit was placed and sealed in a plastic shipping bag and tied with a ribbon to look like a gift. The shipping bags were then placed inside a shipping box that was also labeled with the participant ID number. As participants were enrolled and assigned a study ID number, the assigned box was labeled with a shipping label and the tracking number was recorded. We developed a Zoom training protocol for our team to prepare them to meet with participants.

#### Zoom Meeting Preparation

It was important that the research staff were familiar with and comfortable with Zoom and interacting with participants remotely. Each staff member was sent a HBPM, a HbA_1c_ kit, and a standardized script to follow for the Zoom meetings. Staff meetings were held (via Zoom) to practice instructing participants on how to complete the blood glucose and blood pressure measurements. Each staff member practiced how to instruct participants at least 3 times before they were ready for participant interaction, and everyone received feedback on how to improve their technique and the clarity of their instructions.

After being trained, “mock” Zoom calls were scheduled where staff would guide a participant (another team member) through all of the steps of a study visit. During the mock call, the staff could ask questions and had the option to complete a second mock call if needed. All staff felt comfortable after completing 1 mock call.

#### Step 5: Implementing the Protocol Changes

While the staff prepared to complete the Zoom calls, we launched our Facebook campaign. Advertisements for the study were displayed on our lab’s Facebook page. After viewing the advertisements, individuals who were interested in learning more and potentially participating clicked a link that directed them to a web-based eligibility survey on the Qualtrics platform [33]. The Qualtrics survey contained a study overview, consent document, and eligibility screening questions.

Individuals who provided informed consent to participate in the study and met eligibility criteria were asked to submit their contact information and were contacted by a member of the research team. During a phone call with each potential study participant, the team member verified the information from the Qualtrics screener and administered the Montreal Cognitive Assessment (MoCA) [32]. Given that the test would be administered by phone, the researchers used the blind version of the test that did not incorporate any of the written parts of the traditional MoCA. Participants had to score an 18 or above on the MoCA to be eligible to continue study participation.

#### Step 6: Mitigating Challenges

There were some issues (technical and shipping) that arose during the implementation of the revised BRAINS protocol.

#### Technical Difficulties

Participants experienced some technical difficulties participating in the study remotely. Some participants had some difficulty with Zoom, perhaps because of different levels of experience with the platform. Some participants required more assistance than others, but all were able to measure their blood pressure. The TestMyBrain assessment could not be completed with a smartphone, as the participants’ reaction time information could not be properly collected. This prevented 5 individuals from participating in the study, as we could only enroll those who could complete the study on a tablet or a computer. Even so, during the study, we also found that the “Amazon Fire tablet” was not compatible with the TestMyBrain website.

#### Shipping Issues

There were also some issues with mailing the study kits to participants. Occasionally, a participant’s study materials would not reach the participant’s home in time for their Zoom appointment, which required a research staff member to drive the kit out to the participant and leave it outside to maintain social distancing. The HbA_1c_ tests were sent directly to the DTIL laboratory in Georgia by the participant via the US Postal Service. When the DTIL laboratory contacted our team and stated that a HbA_1c_ test did not arrive, we had to contact participants and ask if they would be willing to retake the test.

#### Step 7: Evaluating Protocol Implementation

After completing the BRAINS study, we reflected on its implementation to evaluate our processes. There were several successes in this revised protocol. Since we were unable to access the fMRI laboratory, we used the additional funds to increase our sample size. Given that the study was conducted remotely, we were able to reach more women in our population of interest, including those who were potentially unable to come in person to participate in our study. Using Zoom, we were able to meet with our participants in their homes, where they were most comfortable, which gave the study a less “clinical feel.” In fact, the principal investigator prepared a 3-minute video thanking participants for joining the study and reminding them how important their participation was. Many of the participants stated that they enjoyed the video and felt at ease after watching it.

## Results

**Figure 1:**
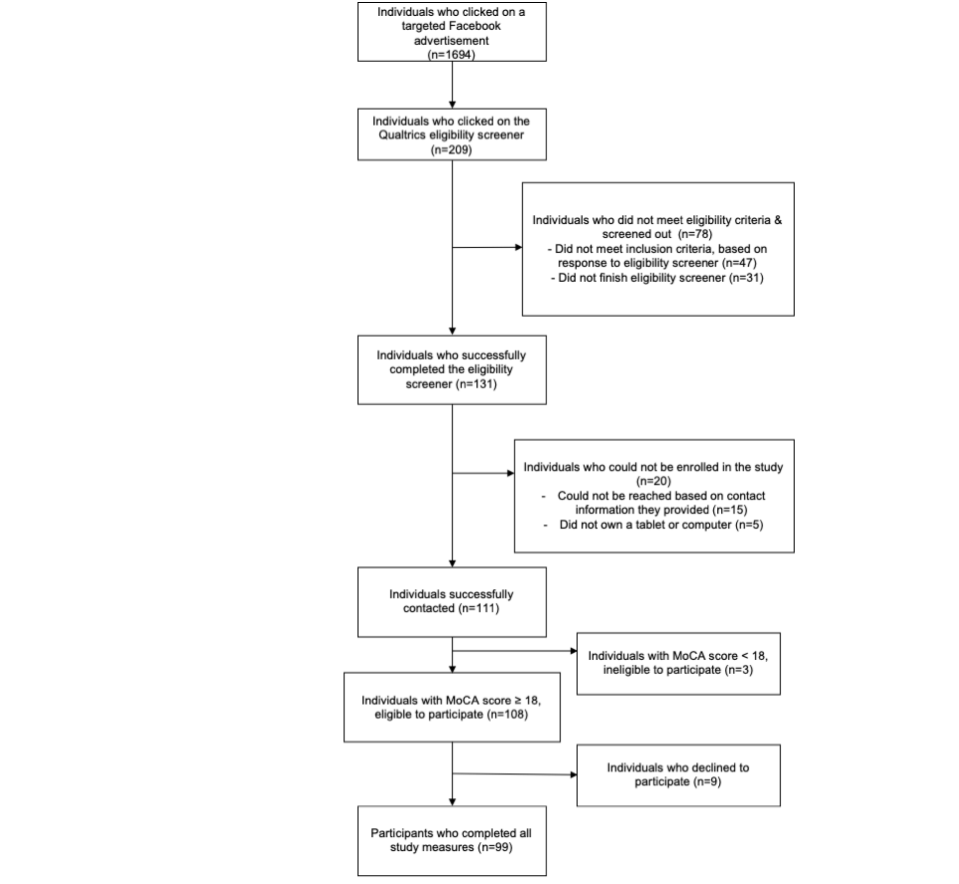
Participant Flow Diagram.

In response to our Facebook campaign, 1694 individuals clicked on the link that directed them to an eligibility screener. Of those who clicked, 131 individuals completed the screener and were eligible to participate in the BRAINS study. Figure 1 illustrates participant enrollment. We conducted our first Zoom appointment in July 2020 and completed our last Zoom appointment in September 2020. Despite the challenges that we faced, we were able to obtain all study measures from 99 participants in a 3-month period. A total of 14 of the HbA_1c_ tests were not received by the DTIL laboratory. When we contacted participants to ask if they would retake the HbA_1c_ test, only 8 participants agreed. Therefore, 6 HbA_1c_ results are missing; this is the only measure that is missing from our data set.

## Discussion

### Overview

Due to the COVID-19 pandemic, we revised our BRAINS protocol from a study designed to be conducted in person to complete remote data collection. We increased our sample size from 50 to 100 participants and were successful in collecting all study measures from 99 Black women with hypertension. In this report, we highlighted the revisions we completed, described how we moved from in-person data collection to remote data collection, and outlined the challenges that needed to be mitigated to reach our goals.

Most importantly, we were able to safely conduct our study, despite the pandemic and associated challenges of in-person, health-related interactions [1]. We reviewed previous studies that had been conducted remotely with videoconferencing as we revised our protocol [17,18]. We designed the revised study so that we could still meet the study aims while ensuring that there were no in-person interactions between the members of our research team and our participants.

An important part of our protocol was to assess the diabetes status among our participants. Although hypertension and diabetes frequently co-occur [8], there is a gap in the literature regarding when Black women with hypertension are most likely to be diagnosed with type 2 diabetes and possible ways to prevent the transition to a diabetes diagnosis. Our protocol is one of the first that outlines steps to assess diabetes status among Black women with hypertension remotely.

Given concerns about self-reported measures [4,5], it was a top priority for our team to determine how to obtain accurate blood pressure measurements. Previous studies have validated HBPM for use outside of the clinic and patient use [35]. Each member of our research team received a HBPM and training on how to use it correctly to measure their blood pressure. After completing the training successfully, our staff was able to guide our participants in applying the blood pressure cuff and measuring their blood pressure. Additionally, while completing the screener to determine eligibility to participate, potential participants had the opportunity to self-report their blood pressure. In our data analysis, we will be able to compare self-reported blood pressure to actual blood pressure measurements.

Some of the measures outlined in the original protocol were unable to be collected remotely. For example, there was no proxy for conducting an fMRI scan, an important part of the original BRAINS protocol. As of this writing, we are inviting participants to undergo an fMRI scan and to repeat some of the original study measures, including the TestMyBrain cognitive assessment, allowing us to examine relationships between the fMRI scan and the neurocognitive battery of tests.

### Limitations

As with any research protocol, there were limitations to implementing our protocol that are worth discussing. Some of the measures that we collected required that participants complete them on a tablet or computer rather than a cell phone. This means that participants who did not own the necessary equipment were automatically excluded from participating in the study, creating bias. During 1 Zoom study visit, we learned that the Amazon Fire tablets were not able to load the TestMyBrain software. Additionally, this study was conducted during the COVID-19 pandemic, so participants’ habits might have been patterned differently than their usual habits. Despite these limitations, this revised protocol was a unique opportunity to safely reach our population of interest during the pandemic.

### Conclusions

The COVID-19 pandemic required revisions of many research protocols for the safety of participants and research staff alike. The pandemic provided a unique opportunity for researchers to gain knowledge about alternate ways of engaging with participants during a “Stay at Home” order. Before the COVID-19 pandemic, the BRAINS protocol was designed so that participants came in person to complete study activities (eg, surveys, serum sample collection, and fMRI). During the COVID-19 pandemic, we pivoted to a completely web-based format to collect survey data, an at-home HbA_1c_ test, and blood pressure measurement. Since we were unable to conduct the fMRI scans, the cost of our research activities dropped drastically, so we were able to enroll additional participants in our study. In addition, working remotely allowed our team to be more flexible with scheduling participants and offer evening and weekend appointments, as we were not subject to the fMRI laboratory’s hours. In the future, researchers can use this study as a guide to develop protocols to reach diverse groups, such as individuals who are unable to participate in research activities due to distance, time, or travel constraints.

## Abbreviations

BRAINS: Brain Relationships Among Information, Neuroprocessing, and Self-Management
fMRI: functional magnetic resonance imaging
HbA_1c_: hemoglobin A_1c_
HBPM: home blood pressure monitor
IRB: institutional review board
MoCA: Montreal Cognitive Assessment

## Data Availability

Data sharing not applicable, as no data were analyzed as of writing this report.
